# Statistical Methods to Adjust for Treatment Switching in Real‐World Clinical Studies: A Scoping Review and Descriptive Comparison

**DOI:** 10.1002/cpt.70013

**Published:** 2025-07-23

**Authors:** Romain Jonathan Collet, Ângela Jornada Ben, Anita Natalia Varga, Frank van Leth, Mohamed El Alili, Jonas Esser, Judith Ekkina Bosmans, Johanna Maria van Dongen

**Affiliations:** ^1^ Department of Rehabilitation Medicine Amsterdam UMC Location Vrije Universiteit Amsterdam Amsterdam The Netherlands; ^2^ Department of Epidemiology and Data Science Amsterdam UMC Location Vrije Universiteit Amsterdam Amsterdam The Netherlands; ^3^ Amsterdam Movement Sciences Musculoskeletal Health Amsterdam The Netherlands; ^4^ Department of Health Sciences Faculty of Science, Vrije Universiteit Amsterdam Amsterdam The Netherlands; ^5^ Department of Health Sciences, Faculty of Science Vrije Universiteit Amsterdam, Amsterdam Public Health Research Institute Amsterdam The Netherlands; ^6^ National Institute for Public Health and the Environment Bilthoven The Netherlands

## Abstract

Real‐world data from sources, such as patient registries and electronic health records, can complement randomized controlled trials by providing timely, generalizable insights that better reflect routine clinical practice. However, the absence of randomization can introduce bias, particularly when treatment switching—defined as deviation from or discontinuation of the initial treatment—is influenced by time‐varying confounders, that is, variables that are associated with both treatment decisions and outcomes over time. This study presents a comprehensive overview of statistical methods used to adjust for treatment switching in real‐world studies to improve causal inference. We systematically searched MEDLINE and Embase for studies comparing at least two statistical methods for adjusting for treatment switching, from inception to December 2024. Forty‐five studies were included, identifying four main categories of methods: (1) traditional approaches (intention‐to‐treat, per‐protocol, as‐treated, repeated measures); (2) propensity score‐based methods (adjustment, matching, marginal structural models); (3) g‐methods other than marginal structural models (g‐computation, structural nested models, longitudinal targeted maximum likelihood estimation); (4) methods addressing unmeasured confounding (regression calibration, instrumental variables). Traditional methods are straightforward, but often yield biased estimates in the presence of treatment switching. Advanced methods, such as g‐methods, are designed to adjust for time‐varying confounding and can produce less biased estimates, though they require complex modeling. Instrumental variables and regression calibration relax the assumption of no unmeasured confounding, but rely on strong, often untestable conditions. By evaluating each method’s assumptions, strengths, and limitations, we support applied researchers in selecting appropriate methods to strengthen causal inference in real‐world studies.

**Study Highlights**

**WHAT IS THE CURRENT KNOWLEDGE ON THE TOPIC?**

Treatment switching, defined as a deviation from or discontinuation of the initially prescribed treatment, is common in real‐world clinical studies. It can introduce confounding bias when influenced by variables that affect both treatment decisions and outcomes over time. While various statistical methods have been developed to address this issue, comprehensive evaluations and comparisons of these methods in the context of RWD remain limited.

**WHAT QUESTION DID THIS STUDY ADDRESS?**

This study systematically reviewed and compared statistical methods developed to adjust for treatment switching in RWD studies, highlighting their assumptions, strengths, and limitations to guide researchers in method selection.

**WHAT DOES THIS STUDY ADD TO OUR KNOWLEDGE?**

We identified 45 studies and organized the statistical methods into four categories: (1) traditional analysis approaches (e.g., intention‐to‐treat and as‐treated), (2) propensity score‐based methods (e.g., adjustment/matching and marginal structural models), (3) g‐methods other than marginal structural models (e.g., g‐computation and longitudinal targeted maximum likelihood estimation), and (4) methods addressing unmeasured confounding (i.e., instrumental variables and regression calibration). This review clarifies when and how each method may be most appropriately applied in RWD studies involving time‐varying treatment patterns.

**HOW MIGHT THIS CHANGE CLINICAL PHARMACOLOGY OR TRANSLATIONAL SCIENCE?**

By critically evaluating statistical methods for handling treatment switching in real‐world clinical studies, this review supports more accurate causal inference. It enables researchers to make better‐informed decisions when analyzing treatment effects, thereby enhancing the validity and real‐world applicability of findings in clinical pharmacology and translational science.

## BACKGROUND

Historically, randomized controlled trials (RCTs) have been the gold standard for estimating causal effects because of their ability to mitigate confounding bias through randomization.^1^, ^2^ Conducted in controlled environments with strict criteria and standardized protocols, RCTs ensure high internal validity. However, RCTs often do not fully reflect the complexities of real‐world clinical practice, limiting their generalizability. As a result, the outcomes of RCTs may only apply to specific patient groups and/or settings, raising questions about broader applicability.^3^, ^4^, ^5^ This creates a gap between interventions’ controlled efficacy, as estimated in RCTs conducted under tightly controlled conditions with strict inclusion criteria, and their real‐world effectiveness, which reflects patient outcomes across broader, more heterogeneous populations.^6^ Real‐world data (RWD) offers a valuable opportunity to bridge this gap by complementing RCTs with timely and generalizable insights that better reflect real‐world clinical practice.^7^ The use of RWD in clinical research has gained popularity in recent decades, driven by its increasing availability and by advances in computational power that allow for the processing and analysis of large, complex datasets. Additionally, advances in internet access, wearable devices, electronic health records, and other e‐health platforms have led to the collection of vast amounts of routinely gathered patient and healthcare data.^8^, ^9^

The observational nature of RWD poses challenges related to confounding bias, since treatment selection and outcomes often depend on patient and clinical characteristics in the absence of randomization.^10^ To address this issue, statistical methods, such as propensity score matching and g‐computation, have been developed to adjust for baseline confounders, producing more accurate causal estimates than unadjusted analyses.^11^ However, an additional layer of complexity arises when treatment is no longer a single‐point exposure, but evolves over time when patients deviate from or discontinue their initial treatment protocols. This phenomenon, known as treatment switching, can introduce further confounding bias if switching is influenced by time‐varying factors that are also associated with the outcomes and may themselves be affected by earlier treatment, such as intervention tolerability, adverse effects, adherence, or disease progression.^12^ To accurately estimate causal effects in analyses using RWD, it is important to use valid methods to adjust for such time‐varying confounders.

Despite the growing body of literature on methods to adjust for treatment switching in RWD studies, comprehensive and critical overviews of these methods remain scarce, with only a few reviews having examined statistical approaches for adjusting for time‐varying confounding in observational settings.^13^, ^14^ However, these reviews did not specifically focus on methodological studies, that is, simulation studies and empirical methodological studies using RWD to assess and compare the performance of different methods for handling treatment switching. Instead, they included a broad set of studies that used or mentioned these methods, such as clinical studies, without critically evaluating their assumptions, strengths, and limitations. Moreover, they did not explicitly focus on RWD, further highlighting the gap in the literature.

Therefore, this study aims to provide a comprehensive overview and rigorous assessment of statistical methods and strategies employed to adjust for treatment switching in RWD studies, regardless of intervention type or medical condition. In addition to systematically identifying these methods, we critically evaluate their assumptions, strengths, and limitations, providing researchers with practical guidance on selecting the most appropriate approach for their specific study contexts.

## METHODS

The scoping review was reported using the Preferred Reporting Items for Systematic reviews and Meta‐Analysis (PRISMA) extension for Scoping Reviews checklist.^15^ A review protocol was developed prior to conducting the review but was not published or registered; it is available upon request from the corresponding author.

### Search strategy

An experienced medical information specialist conducted searches in Medline and Embase from inception until December 2024. The exact search strategy is presented in **Appendix** **S1**. The search strategy used several combinations of index terms and respective keywords, such as treatment switching, crossover studies, observational, etc. Relevant papers’ references were checked for potential additional articles. We also performed searches in Google Scholar using the snowball method (i.e., screening related articles and articles citing the included records).^16^

### Eligibility criteria

Studies were eligible if they compared different methods to account for treatment switching when assessing the causal effect(s) of interventions on patient outcomes using non‐randomized (observational) data, including data from cohort, case–control, or crossover study designs. Treatment switching was defined as any deviation from, or discontinuation of, the initially received treatment, as outlined in **Appendix** **S2**. A detailed definition of treatment switching is provided in **Appendix** **S2**. We included both simulation studies and empirical methodological studies, that is, studies using RWD to assess and compare the results of different methods, regardless of the intervention received by the participants. Furthermore, letters, commentaries, conference abstracts, editorials, and brief communications were excluded, as they likely lacked sufficient information regarding the statistical approaches employed for handling treatment switching.

### Study selection

We imported retrieved records into Rayyan (http://rayyan.qcri.org) for the initial screening of abstracts and titles. After duplicate removal, four researchers (RC, AJB, JvD, ME) screened the title and abstract of 10 articles together to ensure consistency between reviewers. The four researchers then independently screened the title and abstract of the remaining records in pairs of two. Consensus meetings were regularly held throughout the screening process to resolve disagreements. Reasons for excluding articles were recorded and reported, and the search results and study inclusion process were reported and presented in a PRISMA flowchart.^17^

### Data extraction

A data extraction form was developed using Microsoft Excel.^18^ Extracted data fields included the study design, intervention type, statistical methods or strategies employed to adjust for treatment switching, their advantages and disadvantages, and stated assumptions. The extraction form was tested by four reviewers (RC, AJB, JvD, ME) for one paper to ensure consistency and reliability of the data extraction process. After this pilot test, the remaining articles were divided equally, and data were independently extracted in pairs of two reviewers. Consensus meetings involving the four reviewers and three researchers not involved in the screening process (JB, JE, FvL) were held to resolve disagreements.

### Data analysis and synthesis

Statistical methods and strategies for handling treatment switching were narratively summarized and presented in a table summarizing all extracted data (i.e., the method’s name, the studies in which it was assessed, the assumptions it relies on, and its advantages and disadvantages). We extracted and synthesized all methods compared in the included studies, even if not explicitly intended for adjusting treatment switching, to provide a comprehensive overview of current practice.

## RESULTS

### Literature search and study selection

**Figure 1:**
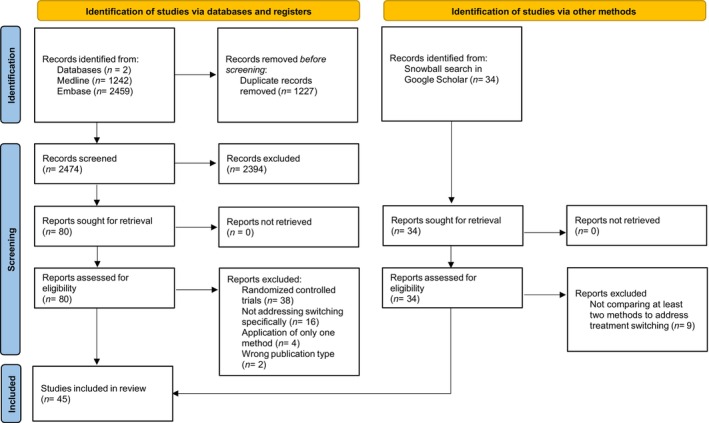
PRISMA flow diagram.

After removing duplicates, the titles and abstracts of the remaining records (*n* = 2474) were screened, resulting in 80 studies (**Figure** **1**). Sixty records were excluded based on full‐text screening and 20 remained.^19^, ^20^, ^21^, ^22^, ^23^, ^24^, ^25^, ^26^, ^27^, ^28^, ^29^, ^30^, ^31^, ^32^ The most common reasons for exclusion were studies focusing on methods for RCTs rather than RWD studies or only applying one method rather than comparing methods to handle treatment switching. Twenty‐five additional studies were identified via reference checking and snowball search in Google Scholar.

### Results of the synthesis

In the 45 included studies, we identified four types of approaches for dealing with treatment switching in RWD studies:

1. Traditional analysis approaches, including intention‐to‐treat, per‐protocol analyses, as‐treated, and repeated measures analyses (using time‐varying treatment indicators).
2. Propensity score‐based methods, including propensity score adjustment, propensity score matching, marginal structural models, and sequential Cox analysis.
3. G‐methods other than marginal structural models, including g‐computation, structural nested models, and longitudinal targeted maximum likelihood estimation.
4. Methods accounting for unmeasured confounding, such as regression calibration and instrumental variable approaches.

We developed these categories based on commonly used terminology in the applied epidemiological literature. Propensity score‐based methods were grouped into the same category based on their reliance on estimated treatment or censoring probabilities to address measured confounding. G‐methods (excluding marginal structural models) were grouped into the same category based on their explicit modeling of time‐varying treatment and confounders. Lastly, regression calibration and instrumental variable approaches were grouped into the same category because of their distinct aim to adjust for bias from unobserved variables, relying on external information or instruments. While some methods could fit multiple categories, we prioritized conceptual clarity and practical relevance in classification.

**Figure 2:**
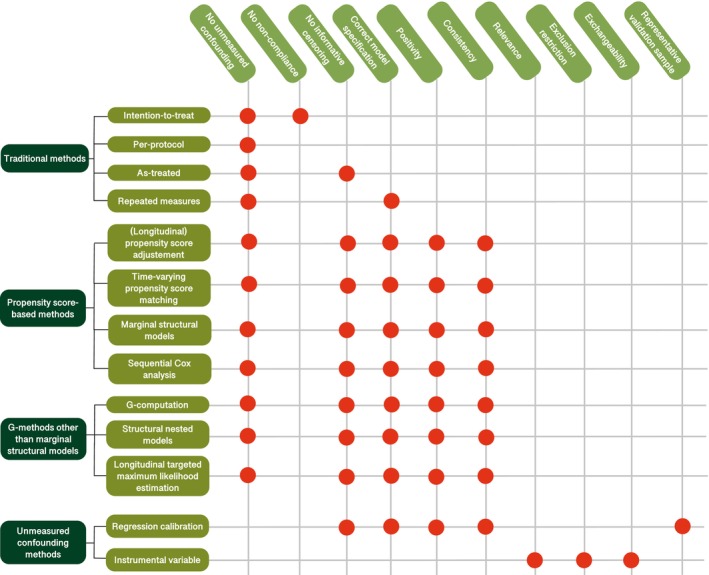
Summary of identified methods and their assumptions. Each row represents a statistical method, and each column lists an assumption that must be met for the method to produce valid results. A red dot means that the method relies on (i.e., assumes) the corresponding assumption. If the assumption is violated in a given study (e.g., unmeasured confounding is present), the method may yield biased results.

**Table** **1** and **Figure** **2** summarize all approaches, their assumptions, advantages, and disadvantages as reported in the included studies. The approaches are discussed in more detail below.

**Table 1:** Summary of identified methods: assumptions, strengths, and limitations

Method	Studies	Assumptions	Advantages	Disadvantages
Traditional analysis methods				
Intention‐to‐treat	Faries *et al*., Belviso *et al*., Danaei *et al*.	No unmeasured confounding, correct model specification, no deviations from initial treatment protocol, no non‐compliance	Includes all participants in the analysis, straightforward to implement and interpret	Does not account for treatment switching, thus potentially yielding biased estimates
Per‐protocol	Faries *et al*.	No unmeasured confounding, correct model specification, adherence to assigned treatment	Estimates treatment effect for compliant participants, straightforward to implement and interpret	Excludes non‐compliant participants, potentially introducing bias; may not reflect real‐world situations
As‐treated	Belviso *et al*., Danaei *et al*., Baek *et al*., Ali *et al*., Cui *et al*., de Keyser *et al*., Suttorp *et al*., Karim *et al*.	No unmeasured confounding, correct model specification, no informative censoring	Reflects treatment effect based on actual treatment received, straightforward to implement and interpret	Sensitive to bias from informative censoring if deviation from initial treatment protocol is outcome‐related
Repeated measures	Faries *et al*., Suarez *et al*., Cole *et al*., He *et al*., Hernán *et al*., Keogh *et al*.	No unmeasured confounding, correct model specification, correct specification of the correlation structure	Provides treatment effects across multiple time points	Potential bias if previous treatment history is related to outcomes and time‐dependent variables
Propensity score‐based methods				
(Longitudinal) propensity score adjustment	Danaei *et al*., Belviso *et al*., Faries *et al*., Ali *et al*.	Positivity, no unmeasured confounding, no informative censoring, correct model specification, consistency	Reduces bias due to baseline and/or time‐varying confounders; easy to implement; Captures treatment effects over time, allowing for modeling treatment changes	May introduce bias if only baseline confounders are included in the analysis
Time‐varying propensity score matching	Lu *et al*., Richey *et al*., Weymann *et al*.	Positivity, no unmeasured confounding, no informative censoring, correct model specification, consistency	Reduces bias due to baseline and time‐varying confounders	May introduce bias if prior treatment is related to future covariates
Marginal structural models	Belviso *et al*., Danaei *et al*., Faries *et al*., Graffeo *et al*., Kim *et al*., Suarez *et al*., Szmulewicz *et al*., Baek *et al*., Ali *et al*., Cui *et al*., de Keyser *et al*., Suttorp *et al*., Karim *et al*., Cole *et al*., Hernán *et al*., Keogh *et al*., Brumback *et al*., Taylor *et al*., Hogan *et al*., Gran *et al*., Kreif *et al*., Lau *et al*., Neugebauer *et al*., Petersen *et al*., Ray *et al*., Saarela *et al*., Schnitzer *et al*., Takeuchi *et al*., Xiao *et al*., Young *et al*., Zheng *et al*.	Positivity, no unmeasured confounding, no informative censoring, correct model specification, consistency	Estimates causal effects while accounting for switching and informative censoring; Includes baseline and time‐varying confounders	Requires detailed tracking of treatment histories and time‐updated covariates; Sensitive to extreme weights
Sequential Cox analysis	Suttorp *et al*., Karim *et al*., Gran *et al*.	Positivity, no unmeasured confounding, no informative censoring, correct model specification, consistency	Estimates causal effects while accounting for switching and informative censoring; Easy to implement; More stable weights than MSMs with IPTW	May introduce bias if treatment switching affects the outcome
G‐methods other than marginal structural models				
G‐computation	Brumback *et al*., Kawahara *et al*., Spieker *et al*. (2018, 2020), Kreif *et al*., Schnitzer *et al*., Schomaker *et al*.	Positivity, no unmeasured confounding, no informative censoring, correct model specification, consistency	Estimates causal effects under multiple treatment scenarios; Can account for time‐dependent confounders; Robust in simulations	
Structural nested models	Brumback *et al*., He *et al*.	Positivity, no unmeasured confounding, no informative censoring, correct model specification, consistency	Estimates causal effects at each time point; Accounts for time‐varying confounders	More complex to implement than g‐computation, may produce biased estimates with small sample sizes
Longitudinal targeted maximum likelihood estimation	Kreif *et al*., Neugebauer *et al*., Petersen *et al*., Schnitzer *et al*., Schomaker *et al*.	Positivity, no unmeasured confounding, no informative censoring, correct model specification, consistency	Accounts for time‐varying confounders; Produces more accurate estimates than g‐computation or MSMs; Robust to misspecification	Complex to implement
Methods accounting for unmeasured confounding				
Instrumental variables	Cui *et al*., Hogan *et al*.	Instrumental variable is independent of survival outcomes and confounders For instrumental variable‐based MSM: Positivity, no unmeasured confounding, no informative censoring, correct model specification, consistency	Does not rely on the no unmeasured confounders assumption	Challenging to identify an adequate instrumental variable which is independent from switching and outcomes; The validity of the instrumental variable cannot be empirically tested
Regression calibration	Burne *et al*.	Positivity, no unmeasured confounding, no informative censoring, correct model specification, consistency	Does not rely on the no unmeasured confounders assumption	Representative validation samples containing data on all possible confounders are often not available

#### Traditional analysis approaches

Traditional analysis methods include intention‐to‐treat, per‐protocol, as‐treated, and repeated measures analyses.^20^, ^21^, ^29^, ^33^, ^34^, ^35^, ^36^, ^37^, ^38^, ^39^, ^40^, ^41^, ^42^ Although intention‐to‐treat and per‐protocol analyses do not explicitly adjust for treatment switching, they were assessed as comparators in the included studies and are reported here for completeness. All traditional analysis methods assume no unmeasured confounding and correct model specification.

##### Intention‐to‐treat analysis

###### Method overview

The intention‐to‐treat approach includes all participants in the statistical analysis, retaining them in their initially assigned treatment groups regardless of discontinuation or switching. Three included studies evaluated the intention‐to‐treat approach, primarily using it as a comparator rather than as a method to adjust for treatment switching.^19^, ^20^, ^21^ Within those studies, ANCOVA or regression models were used to estimate average treatment effects.

###### Assumptions, strengths, and limitations for handling treatment switching

While intention‐to‐treat is relatively straightforward to implement across diverse settings (e.g., different outcomes of interest), this method assumes no non‐compliance or deviations from the assigned treatment.^19^ As a result, the effects of subsequent treatments are attributed to the original treatment, potentially introducing bias and misrepresenting the true effectiveness of the treatment under study.^19^, ^21^

##### Per‐protocol analysis

###### Method overview

A per‐protocol analysis estimates the effect of a treatment by including only participants in the statistical analysis who strictly adhered to their assigned treatment throughout the study, and hence excluding all participants who switched treatments or discontinued participation. Only one study by Faries *et al*.^21^ evaluated this approach, using it as a comparator rather than as a method for adjusting for treatment switching. In this study, ANCOVA models were applied to estimate the average treatment effect among the per‐protocol population.

###### Assumptions, strengths, and limitations for handling treatment switching

A significant drawback of this method is the potential introduction of selection bias, as adherent participants often differ systematically in their characteristics compared to non‐adherent participants.

##### As‐treated analysis

###### Method overview

The as‐treated approach analyzes each participant’s data only as long as they remain on their initial treatment, and data collected after treatment switching are excluded from the statistical analyses. This approach was evaluated in eight studies.^19^, ^20^, ^33^, ^34^, ^36^, ^37^, ^40^, ^42^ Within those studies, effect estimates were derived using Cox proportional hazards models to evaluate time‐to‐event outcomes based on actual treatment received.

###### Assumptions, strengths, and limitations for handling treatment switching

Through simulations, Danaei *et al*.^20^ and Belviso *et al*.^19^ found that the results from such an as‐treated approach were similar to those of the intention‐to‐treat approach in scenarios with minimal treatment switching. However, in the presence of substantial treatment switching, the as‐treated approach is prone to bias due to informative censoring.^36^, ^37^, ^40^

##### Repeated measures analysis

###### Method overview

While not designed to adjust for treatment switching in a causal framework, repeated measures analyses allow researchers to model evolving treatment patterns and their associations with repeated outcome measures. Six studies evaluated this approach.^21^, ^29^, ^35^, ^38^, ^39^, ^41^ For example, Faries *et al*.^21^ divided data into episodes (e.g., a year divided into four 3‐month periods). Then, ANCOVA was performed for each episode with the outcome, defined as change from the previous visit, as the dependent variable. The model included baseline covariates and a variable indicating whether the participant switched treatments during that episode. Least squares mean treatment differences from each episode were then summed to provide an overall treatment difference, with confidence intervals estimated with bootstrapping. Five other studies applied repeated measures using generalized estimating equation (GEE) models, incorporating baseline covariates and the treatment received at the beginning of each period as independent variables.^29^, ^35^, ^38^, ^39^, ^41^

###### Assumptions, strengths, and limitations for handling treatment switching

An advantage of a repeated measures analysis is that it provides treatment effects across multiple time points and can, therefore, account for treatment switching. However, a repeated measures analysis can produce biased estimates if important time‐varying confounders are omitted.^38^, ^39^, ^41^ Bias may also arise if treatment history influences both subsequent outcomes and other time‐dependent variables that are not properly accounted for in the model. Keogh *et al*.^41^ extended the GEE method by adjusting for past exposures, outcomes, and time‐varying covariates, which they found to result in more precise effect estimates than the standard GEE models that do not adjust for time‐varying covariates and past treatment or outcome history. GEE models rely on an assumed correlation structure, such as one that presumes equal correlation among observations within a subject (i.e., exchangeable correlation structure) or one that allows correlations to differ depending on the time lag between observations (i.e., autoregressive correlation structure). However, this structure is assumed rather than estimated from the data, and if it is misspecified, it can lead to incorrect standard errors and potentially misleading conclusions.^29^, ^38^, ^39^

#### Propensity score‐based methods

Propensity score (PS) methods include (longitudinal) PS adjustment and propensity score matching. These methods aim to reduce confounding by balancing treatment groups with respect to observed covariates, using estimated propensity scores, that is, the predicted probability of receiving a given treatment based on those covariates. Propensity scores are typically estimated using logistic regression with treatment assignment as the dependent variable and observed baseline confounders as covariates. PS methods rely on five key assumptions^20^, ^21^, ^25^, ^27^, ^28^:

1. Positivity: There is a non‐zero probability of being assigned to the treatment for every combination of covariates.
2. No unmeasured confounders: All variables associated with treatment assignment and outcome are measured and/or adjusted for. At each time point, treatment assignment or censoring is independent of the potential outcomes given the observed history up to that time.
3. No informative censoring: Conditional on the measured confounders, censoring is unrelated to the treatment and outcomes.
4. Correct model specification: The model includes all appropriate variables and correctly specifies functional relationships (e.g., quadratic or logarithmic).
5. Consistency: The potential outcome of an individual under a given treatment is the outcome that will actually be observed for that individual.

##### (Longitudinal) propensity score adjustment

###### Method overview

PS adjustment was assessed in four studies.^20^, ^21^, ^34^, ^43^ In the study by Danaei *et al*.,^20^ the observations of all participants were segmented into so‐called “person‐trials.” That is, each participant was treated as a new observation whenever they deviated from their initial treatment, with covariate values for each person‐trial being based on the most recently recorded data before the start of that “person‐trial”. Propensity scores were then estimated for each “person‐trial,” and a pooled logistic regression model was then fitted with indicators for treatment initiation and quantiles of the estimated PS.^20^ Because participants contributed to multiple “person‐trials,” robust (sandwich) variance estimators were used to account for clustering within individuals. The average hazard ratio found through this method was similar to the intention‐to‐treat approach.

Faries *et al*.^21^ proposed a longitudinal framework for handling treatment switching. They divided the participants’ follow‐up period into episodes, with each episode corresponding to the duration that a participant remained on a specific treatment. These episodes were then analyzed using a mixed‐effects model, including PS bins as a covariate. PS binning involves grouping participants with similar propensity scores into categories. This approach yielded treatment effect estimates consistent with those from other causal inference methods, such as marginal structural models, whereas the intention‐to‐treat analysis in the same study produced markedly different estimates that failed to account for treatment switching.

Finally, Ali *et al*.^34^ and Brumback *et al*.^43^ added time‐varying propensity scores directly as covariates to their regression models (Cox model and ordinary least squares models, respectively) for treatment effect estimation. The hazard ratios obtained using this approach were consistent with those from marginal structural models and g‐computation. Unlike traditional PS‐adjustment based on baseline covariates alone, these models accounted for time‐varying covariates by updating propensity scores at subsequent time points.

###### Strengths and limitations for handling treatment switching

An advantage of PS‐adjustment is that it is relatively easy to implement and reduces bias due to measured confounders. However, when based solely on baseline covariates, this method does not address bias introduced by treatment switching. In addition, results may be biased if either the PS model or the outcome model is not correctly specified, or in the presence of time‐dependent confounders that are influenced by prior treatment.^34^, ^43^

##### Time‐varying propensity score matching

###### Method overview

Three studies applied time‐varying PS matching to adjust for treatment switching.^44^, ^45^, ^46^ This method pairs treated and untreated individuals with similar propensity scores, aiming to mimic the conditions of an RCT by balancing covariates between groups. Lu^44^ and Weymann *et al*.^46^ estimated time‐varying treatment risk using Cox proportional hazards models, which model the hazard of treatment initiation at each time point based on both baseline and time‐dependent covariates. Although the outputs are not true propensity scores (since hazards are not probabilities), these risk scores served as proxies for an individual’s relative likelihood of treatment initiation. Specifically, the linear predictors from the Cox model were used to match treated and untreated individuals with comparable treatment risk profiles at a given time. Two matching strategies were used:

- Sequential matching: Treated individuals were matched to untreated counterparts within the same risk set, that is, among those who had not yet initiated treatment and were still eligible to do so at that time. This strategy preserves covariate balance over time and prevents the use of future information in the matching process^44^, ^46^;
- Simultaneous matching: Matching was performed across all treated and untreated individuals at once, without constructing time‐specific risk sets.^44^

Both matching methods performed similarly.^44^

Richey *et al*.^45^ extended the aforementioned sequential approach to time‐varying PS by allowing replacement of comparators, meaning that untreated individuals could be matched to more than one treated individual. They also applied calipers (i.e., restrictions on how closely matched pairs could be in terms of PS values) to enhance covariate balance. Their simulation study showed that both sequential matching and its extended version yielded comparable effect estimates.

###### Strengths and limitations for handling treatment switching

While time‐varying PS matching improves covariate balance, as with all PS‐based methods, it requires careful parameter tuning (i.e., manual assessment of covariate balance and appropriate choice of caliper), which can be time‐consuming.^46^ Conversely, simultaneous matching assumes treatment assignment to be independent of future covariates, which may lead to biased estimates if this assumption is violated.^46^

###### Alternative longitudinal matching methods

Beyond PS‐based approaches, alternative longitudinal matching methods were described in three studies.^45^, ^46^, ^47^ These include sequential stratification, which matches participants exactly on covariates rather than propensity scores,^45^, ^47^ and longitudinal genetic matching, a machine learning‐based extension of PS matching that iteratively optimizes covariate balance over time.^46^ These methods demonstrated improved covariate balance and yielded less biased or similarly accurate treatment effect estimates compared to standard PS matching approaches.

##### Marginal structural models

Marginal structural models (MSMs) estimate causal effects at the population level using inverse probability weighting (IPW) to adjust for time‐dependent confounding, providing a marginal summary, such as the average effect of a treatment or a treatment sequence over time. These models are referred to as “marginal” because they estimate the population‐level mean of the potential outcome under a given treatment strategy and “structural” because they specify causal relationships in terms of counterfactual outcomes rather than observed data.^48^ Two approaches were identified: (1) MSMs adjusting for baseline confounding only and (2) MSMs accounting for both baseline and time‐varying confounding.

##### Adjusting for baseline confounding only

###### Method overview

With this approach, inverse probability of treatment weighting (IPTW) assigns a weight to each participant based on the inverse probability of receiving the treatment they initially received (i.e., the inverse of the PS). Specifically, participants who received the treatment are weighted by 1/PS, while those who did not are weighted by 1/(1 − PS). This creates a pseudo‐population in which treatment assignment is independent of measured baseline confounders, effectively balancing the distribution of confounders between treatment groups. This approach was assessed in eight studies that applied IPTW in combination with outcome models, such as ANCOVA,^21^ Cox regression,^19^, ^20^, ^25^, ^32^ Kaplan–Meier estimators,^30^, ^31^ GEE,^29^ and logistic or multinomial regression.^23^, ^26^ These models were used to estimate treatment effects in the weighted samples.

###### Strengths and limitations for handling treatment switching

IPTW has the advantage that it is relatively easy to implement and helps reduce bias due to baseline confounders. However, it does not fully address treatment switching, as it focuses solely on the initial treatment received and assumes that subsequent treatment decisions are independent of the outcome. This, however, may not hold if treatment switching is related to the outcome. Additionally, the method requires relatively large sample sizes and can be sensitive to extreme weights, which may distort the results. To mitigate the impact of extreme weights, methods such as stabilization, normalization, and truncation have been proposed. Although these techniques are not covered in detail in this review, we refer interested readers to published guidance and empirical applications for further information.^49^, ^50^, ^51^

##### Adjusting for baseline and time‐varying confounding

###### Method overview

When longitudinal data are available, inverse probability weights can be calculated at each time point based on treatment and covariate values recorded up to that point, and cumulative weights can then be applied in the MSM. This approach is particularly useful for addressing potential biases in studies with complex treatment patterns, such as those involving treatment switching. Several studies have evaluated this method, extending the regular IPTW approach (see “Adjusting for baseline confounding only”) by incorporating time‐varying inverse probability treatment weights estimated using baseline and post‐baseline confounders.^19^, ^21^, ^30^, ^31^, ^33^, ^34^, ^35^, ^36^, ^37^, ^39^, ^40^, ^41^, ^42^, ^43^, ^47^, ^48^, ^52^, ^53^, ^54^, ^55^, ^56^, ^57^, ^58^, ^59^, ^60^, ^61^, ^62^, ^63^ These time‐varying weights are calculated for each participant, accounting for treatment sequences and measured baseline and time‐varying confounders. Furthermore, some studies calculated the inverse probability of censoring weight (IPCW) to account for bias resulting from informative censoring.^19^, ^22^, ^23^, ^32^, ^33^, ^35^, ^39^, ^40^, ^42^, ^48^, ^54^, ^58^, ^60^ In contrast to IPTW, the denominator of an IPCW represents the probability of a participant remaining uncensored given a set of covariates measured at baseline and post‐baseline (i.e., time‐varying covariates). A final weight can then be obtained by multiplying the IPTW and the IPCW, which simultaneously addresses baseline and time‐varying confounders, as well as selection bias due to informative censoring.^19^, ^22^, ^30^, ^35^, ^39^, ^40^, ^42^, ^48^, ^54^, ^58^, ^60^

###### Strengths and limitations for handling treatment switching

An advantage of MSMs is that they allow for the use of all available data and seem to produce more consistent and accurate estimates compared to alternative methods, such as PS adjustment, which fails to account for post‐baseline confounders.^19^, ^21^, ^29^, ^31^, ^37^, ^54^, ^57^, ^58^ In addition, IPTW estimation corrects for confounding by reweighting observations based on the inverse probability of treatment, enabling the estimation of marginal (population‐average) treatment effects under time‐varying confounding.^43^ Finally, by combining IPTW and IPCW, MSMs can eliminate bias arising from measured (time‐varying) confounders and selection bias due to informative censoring, thus providing more reliable estimates of the effect of the initial treatment.^20^, ^22^, ^33^, ^40^, ^42^, ^58^, ^60^

Despite these advantages, MSMs present several limitations. First, IPTW analysis is susceptible to unstable weights, leading to higher variance estimates than alternative estimation methods, such as g‐computation.^35^, ^36^, ^37^, ^39^, ^40^, ^41^, ^43^, ^47^, ^48^, ^52^, ^53^, ^54^, ^56^, ^57^, ^63^ Second, the estimation of time‐varying weights becomes increasingly complex as the number of treatment switches grows, requiring more detailed tracking of treatment histories and time‐updated covariates.^29^, ^31^, ^58^ Third, MSMs struggle to accommodate interactions between exposure and time‐dependent covariates due to their reliance on weighting rather than direct adjustment.^41^

##### Sequential Cox analysis

###### Method overview

The sequential Cox approach, a method designed to estimate causal treatment effects by mimicking a sequence of RCTs, was employed in three studies.^40^, ^42^, ^52^ This approach divides follow‐up time into intervals, constructing multiple “mini‐trials” based on treatment initiation timing, and employs a Cox model to compare treated individuals with untreated controls within each interval. To prevent confounding, it is necessary to account for the fact that treatment initiation is time‐dependent. To address this, individuals who start treatment later are artificially censored; that is, they are removed from the risk set at the time of treatment initiation to avoid bias due to time‐dependent treatment allocation. However, this artificial censoring introduces the risk of informative censoring, meaning that censoring may depend on patient characteristics associated with the outcome. To mitigate this bias, Cox models are weighted using inverse probability of censoring weights (IPCW), ensuring that the censored individuals are accounted for appropriately in the analysis.

In the study by Gran *et al*.,^52^ results across mini‐trials were pooled using composite likelihood inference, where likelihood contributions from each mini‐trial were combined into a single composite likelihood function. Meanwhile, Karim *et al*.^40^ employed a stratified Cox model, fitting a single stratified Cox regression to the combined dataset rather than pooling separate estimates from each mini‐trial. Suttorp *et al*.,^42^ used a stacking approach, in which datasets from each mini‐trial were merged into a single dataset, and a Cox regression was performed on the stacked dataset to estimate an overall treatment effect.

###### Strengths and limitations for handling treatment switching

One advantage of these sequential Cox approaches is their intuitive framework for estimating treatment effects, while avoiding issues such as unstable weights from MSMs, since IPCW weights tend to be less variable than IPTW weights.^40^, ^52^ However, this method has some limitations, as it does not fully account for treatment switching. This is because it assumes that treatment effects remain consistent across all mini‐trials,^40^ which can be problematic if treatment switching affects the outcome, as the method does not explicitly account for this. Additionally, including the same individuals in multiple mini‐trials can result in underestimated standard errors.^40^, ^42^ Finally, when event rates are low, time‐dependent confounding may not be fully eliminated despite using the sequential Cox approach.^40^

#### G‐methods other than marginal structural models

G‐methods are a family of approaches developed to estimate causal effects, particularly in the presence of time‐varying confounding.^64^ These methods are based on the g‐formula, which uses mathematical models to adjust for confounders and estimate counterfactual outcomes that were not observed in reality. Like PS‐based methods, g‐methods assume positivity, no unmeasured confounding, no informative censoring, and correct model specification. In the context of treatment switching, they can be applied to estimate causal effects under different treatment scenarios.^24^

G‐methods were employed in 11 studies to address treatment switching in RWD settings.^24^, ^27^, ^28^, ^38^, ^43^, ^53^, ^55^, ^56^, ^59^, ^65^ These include: (1) g‐computation, which explicitly models the expected outcome under different treatment scenarios, (2) structural nested models, which iteratively estimate treatment effects while adjusting for time‐dependent confounding, and (3) longitudinal targeted maximum likelihood estimation (LTMLE), a semi‐parametric, doubly robust approach that refines g‐computation by incorporating machine learning techniques for improved efficiency and reduced model misspecification. Doubly robust methods combine two models (one for the treatment assignment (or censoring) and one for the outcome) and yield consistent estimates if at least one of the two models is correctly specified.

##### G‐computation

###### Method overview

The g‐computation algorithm is a statistical method used to estimate counterfactual outcomes in observational studies. It allows for the estimation of treatment effects by modeling the outcome as a function of observed covariates and treatment status. The procedure follows three main steps:

1. Modeling the outcome: a regression model is fitted to estimate the relationship between the observed outcome, treatment, and confounders.
2. Predicting counterfactual outcomes: the estimated model is then used to predict the outcome under different treatment scenarios (e.g., if all individuals had been treated vs. if none had been treated).
3. Averaging to estimate treatment effects: the expected outcomes under each treatment scenario are averaged over the study population, allowing for the estimation of causal effects by comparing the mean predicted outcomes across treatment groups.

Kawahara *et al*.^24^ conducted simulations where treatment was administered at baseline, with the possibility of switching or a second dose on the following day. They used g‐computation to estimate differences in mean outcomes between individuals who received treatment at both time points vs. those who never received it. This method can also be extended to compare other treatment scenarios, such as receiving the treatment only at baseline vs. never receiving treatment. Spieker *et al*.^27^, ^28^ employed an extended version of the g‐computation, the nested g‐computation, which iteratively estimates the outcome while incorporating prior outcomes and death as confounders. Schnitzer *et al*.^59^ employed another variant of g‐computation, the untargeted g‐computation, which uses regression models to predict outcomes based on observed treatment history and confounders. Then, instead of relying on the observed confounder distribution, it recalculates predictions using an adjusted distribution that reflects the treatment scenario of interest. The final estimate is obtained by averaging the predicted outcomes across the modified confounder distribution.

###### Strengths and limitations for handling treatment switching

A major advantage of g‐computation is its ability to predict individual‐level counterfactual outcomes, allowing for a direct estimation of what would have happened for each individual had they received a different treatment regimen, including scenarios with treatment switching.^43^ Furthermore, g‐computation accounts for time‐varying confounders influenced by prior treatment, enabling unbiased estimation under complex treatment scenarios.^24^, ^65^

##### Structural nested models

###### Method overview

Structural nested models provide an alternative framework for handling time‐varying confounding when estimating causal effects in the presence of treatment switching.^38^, ^43^ Unlike g‐computation, which models the expected outcome under predefined treatment scenarios, structural nested models focus on estimating so‐called “blip‐functions”, i.e., the incremental causal effect of receiving treatment at a given time point, conditional on past treatment and covariate history. These models use g‐estimation, a technique that estimates causal parameters by identifying the treatment effect that would make the observed treatment assignment appear unrelated to future outcomes, after adjusting for past covariates and treatment history. Unlike g‐computation, which requires modeling the full conditional expectation of the outcome under each treatment scenario, g‐estimation in structural nested models targets only the parameters of the “blip function” (the causal effect of treatment at a given time, conditional on treatment and covariate history), thereby reducing reliance on the correct specification of the overall outcome model.

###### Strengths and limitations for handling treatment switching

Structural nested models, like g‐computation, allow for explicit modeling of effect modification by time‐varying covariates, enabling researchers to assess how treatment effects evolve over time.^42^ However, they are more complex to implement compared to g‐computation or MSMs, and simulations indicate that they perform well with large samples but may produce biased estimates with smaller samples.^38^, ^43^

##### Longitudinal Targeted Maximum Likelihood Estimation

###### Method overview

Longitudinal Targeted Maximum Likelihood Estimation (LTMLE) is a doubly robust, semi‐parametric method for estimating causal treatment effects in the presence of time‐dependent confounding.^53^, ^55^, ^59^, ^63^ This method was described in five studies.^53^, ^55^, ^56^, ^59^, ^65^ Unlike standard parametric models, LTMLE integrates machine learning techniques, such as the *Super Learner* algorithm, to better capture complex relationships between variables and reduce the risk of model misspecification.^65^ The *Super Learner* algorithm improves predictions by combining multiple models (e.g., logistic regression, lasso regression, random forests) and giving more weight to the most accurate ones. This reduces reliance on a single model and makes the estimates more reliable. LTMLE extends the g‐computation by sequentially modeling the relationship between treatment, confounders, and the outcome over time, incorporating a *targeted update* step that improves estimation by adjusting for treatment assignment.^53^ This approach has been mathematically proven to reduce bias and produce more reliable estimates compared to other methods, such as MSMs.^55^, ^59^

###### Strengths and limitations for handling treatment switching

LTMLE has several methodological advantages. Its double robustness means that it yields valid estimates as long as either the outcome model or the treatment (or censoring) model is correctly specified, but not necessarily both.^55^, ^59^, ^65^ Furthermore, when both models are correctly specified, LTMLE achieves optimal statistical efficiency within the class of semi‐parametric estimators, meaning that it produces more precise effect estimates compared to other semi‐parametric approaches.^55^, ^59^ However, LTMLE can be computationally intensive, especially in studies with long follow‐up periods and many time‐varying confounders, as it requires fitting multiple models for the outcome, treatment, and censoring mechanisms at each time point and when incorporating a large number of Super Learners for estimating these models.^65^ Petersen *et al*.^56^ introduced two modified approaches: Pooled LTMLE and Stratified LTMLE. Pooled LTMLE reduces computational burden by combining data across all time points into a single model, but assumes a constant treatment‐confounder relationship, which may introduce bias if these relationships vary over time. Stratified LTMLE, in contrast, fits separate models for each time point, allowing for time‐specific treatment effects but increasing computational complexity and reducing sample size per time point.

#### Methods accounting for unmeasured confounding

In addition to the methods identified for addressing measured confounding in the presence of treatment switching, three studies evaluated approaches specifically designed to account for unmeasured confounding in the presence of treatment switching, including: (1) Regression calibration^66^ and instrumental variable approaches.^36^, ^48^

##### Regression calibration

###### Method overview

Regression calibration adjusts for unmeasured confounders by treating them as measurement error in the PS.^66^ It uses a validation sample where unmeasured confounders are observed to model the relationship between the error‐prone PS (calculated without unmeasured confounders) and the “gold‐standard” PS (calculated from the validation sample). This correction improves the estimation of true treatment assignment probabilities. Burne and Abrahamowicz^66^ extended this approach by incorporating Martingale residuals (i.e., residuals from a survival model), along with treatment status and measured confounders, as predictors in a multiple imputation model to estimate values for unmeasured confounders. This extension outperformed standard regression calibration, substantially reducing bias. Under the condition that the validation sample is representative of the population, it offers a promising solution for addressing unmeasured confounding.

##### Instrumental variable approaches

###### Method overview

Instrumental variable (IV) approaches do not require a validation sample and instead use an external variable associated with treatment assignment but affecting the outcome only through its influence on the treatment, mimicking randomization and enabling unbiased causal inference.^36^, ^48^ IV‐based MSMs refine IPTW estimation by incorporating a time‐varying IV into the weighting model.^36^ With this approach, traditional two‐stage least squares first predicts treatment assignment using the IV and then substitutes this predicted treatment in the outcome model.^48^

###### Assumptions, strengths, and limitations for handling treatment switching

While IV methods theoretically eliminate confounding bias, identifying a valid and strong IV is challenging because it relies on strong assumptions: (1) *relevance*, meaning the IV must influence treatment allocation; (2) *exchangeability*, meaning the IV should not be associated with the outcome except through its effect on treatment; (3) *exclusion restriction*, meaning the IV must affect the outcome only through the treatment and not through alternative pathways.^67^ This becomes even more complex when using time‐varying IVs, as the assumptions of independence and exclusion restriction must hold at every time point where the instrument varies. Since IV validity cannot be empirically tested, it must be justified solely based on subject matter expertise.^48^

## DISCUSSION

This study provides a comprehensive overview of statistical methods to adjust for treatment switching in RWD studies. We identified four main categories of approaches: (1) traditional methods, such as intention‐to‐treat, per‐protocol, as‐treated, and repeated measures analyses; (2) PS‐based methods, such as PS adjustment or matching, MSMs, and sequential Cox analysis; (3) g‐methods other than MSMs, including g‐computation, structural nested models, and longitudinal targeted maximum likelihood estimation; and (4) methods addressing unmeasured confounding, such as regression calibration and IVs. Several of these methods, particularly MSMs, g‐computation, and LTMLE, align with the framework of target trial emulation, which aims to structure RWD to mimic the design and interpretation of an RCT.^68^

Each of these categories of approaches, and corresponding methods, come with specific assumptions, benefits, and drawbacks. Traditional methods are straightforward but may fail to adequately account for treatment switching, potentially leading to biased estimates. PS adjustment or matching reduces confounding by balancing observed covariates between groups, but relies on the no unmeasured confounders assumption and correct specification of the PS model, which may not hold in RWD settings. Marginal structural models and other g‐methods offer greater flexibility by explicitly modeling time‐varying confounding. However, they require complex modeling and, like most other methods, rely on strong assumptions, such as no unmeasured confounders and the correct specification of both the outcome and the confounder models. Finally, regression calibration and IVs avoid reliance on the assumption of no unmeasured confounders by using a validation sample or mimicking randomization. However, their applicability depends on the representativeness of the validation sample and the validity of the IV, respectively, which may not hold in all RWD settings and cannot be empirically tested. Hence, all of these methods have strengths and limitations, and the choice should be guided by the study context, the nature of the data, and the plausibility of the underlying assumptions.

### Comparison with previous reviews

A prior systematic review investigated various methods for estimating causal effects in the presence of time‐varying confounding, which commonly arises in RWD studies due to treatment switching when changes in treatment are driven by evolving prognostic factors.^13^ The identified methods included (nested) g‐computation, MSMs, and TMLE. This review, however, defined methods *a priori* and then systematically reviewed the extent to which these methods have been used in the literature. In contrast, we undertook a comprehensive scoping review approach to identify all methods available for handling treatment switching, including only methodological studies that directly compared these approaches. This broader perspective allowed us to evaluate and synthesize their relative characteristics, strengths, and limitations.

Another recent scoping review also sought to provide a comprehensive overview of statistical methods for handling time‐varying confounding in RWD studies.^69^ However, its literature search primarily identified review articles, with only 10 original studies. Our more extensive search identified 45 original articles, enabling a detailed comparison of methods and a thorough summary of their advantages and disadvantages. Additionally, while this prior review identified a limited set of methods (mainly longitudinal matching and g‐methods), we identified a broader range, including various PS‐based approaches and advanced machine learning techniques, such as LTMLE, which were not covered. Overall, by providing a more comprehensive and detailed overview of available methods and presenting our findings in a clear and accessible narrative, we offer epidemiologists and researchers practical guidance for selecting the most appropriate methods for specific contexts.

### Challenges, considerations, and practical implications

In RCTs, unmeasured confounding is not typically modeled explicitly, as randomization is expected to balance both measured and unmeasured baseline characteristics between treatment groups. In contrast, in RWD studies, where treatment assignment is not randomized, unmeasured confounding remains a major methodological challenge, particularly in settings involving multiple treatment switches and time‐varying confounders.^25^ While treatment switching can occur in both RCTs and RWD studies due to evolving clinical factors, such as tolerability, disease progression, or patient preference, its implications differ: in RCTs, switching occurs within a controlled, protocol‐driven context where baseline confounding is minimized; in RWD, switching decisions are made in uncontrolled, heterogeneous settings and are often driven by complex, context‐specific factors that are incompletely observed. This makes the confounding introduced by switching harder to adjust for in RWD. Many of the methods identified in this review, such as g‐methods and IV approaches, have also been applied to RCTs.^70^, ^71^ However, their performance and implementation challenges may differ considerably across these settings, particularly because the underlying functional relationships between variables are often more complex and less well‐specified in RWD, in addition to the more frequent occurrence of missing data and incomplete covariate information. As for the latter, many included studies used IPCW to address missingness, in settings where censoring was likely informative due to treatment switching influenced by time‐varying covariates, highlighting IPCW’s potential for mitigating post‐treatment bias in RWD.^19^, ^30^, ^60^ However, none combined treatment switching adjustment methods with other methods to handle missing data, such as multiple imputation. Future research is needed to evaluate and compare such combined strategies, including IPCW‐ and multiple imputation‐based approaches, to clarify their performance and suitability in RWD contexts. We note that meticulous planning and extensive data collection are likely essential for the effective application of advanced methods for handling treatment switching, particularly in the presence of substantial time‐varying confounding and missing data. This involves not only measuring all potential (time‐varying) confounders, but also ensuring that these measurements are performed consistently over time and not too long before treatment switching can occur.

Unfortunately, the quality and completeness of data in RWD sources are often suboptimal, as evidence highlights frequent inaccuracies and incomplete records in electronic health databases (e.g., missing or misclassified entries).^72^, ^73^, ^74^

Among the identified approaches, regression calibration and IVs stand out as they do not rely on the assumption of no unmeasured confounding, making them theoretically appealing for addressing treatment switching in RWD studies. However, the practical application of these methods hinges on the availability of a representative validation sample or a valid IV, which can be highly challenging to identify. For example, IVs must meet stringent assumptions: (1) *relevance*, meaning the IV must influence treatment allocation; (2) *exchangeability*, meaning the IV should not be associated with the outcome except through its effect on treatment; and (3) *exclusion restriction*, meaning the IV must only affect the outcome via its impact on the treatment and not through alternative pathways.^67^ Recent systematic reviews examining the quality and completeness of IV reporting in RWD studies have highlighted that over two‐thirds of studies failed to fully address the assumptions required for a valid IV.^67^, ^75^, ^76^ This underscores the difficulty of identifying and validating appropriate IVs in practice, which restricts their real‐world applicability.

Other identified methods, including PS methods and g‐methods, not only rely on the no unmeasured confounding assumption, but also heavily depend on the correct specification of both the outcome and the confounder models. Kim and Cable^25^ noted that model misspecification becomes particularly problematic in scenarios involving many switches and high levels of missing data. Doubly robust approaches, such as LTMLE, offer greater flexibility, enabling robust estimations even when either the outcome or confounder model is misspecified.^11^ However, unlike MSMs, which have been widely implemented and assessed, their performance has mainly been evaluated in simulation studies with a single‐point exposure.^11^ Future research should assess whether doubly robust methods consistently outperform other approaches in complex datasets with frequent switching and varying levels of missing data, ensuring accurate effect estimation in RWD studies.

Finally, many of the identified methods remain challenging to implement in practice, as they are not readily available in commonly used statistical software packages (e.g., SPSS, STATA). This lack of accessibility can pose a significant barrier for applied researchers, particularly those without advanced programming expertise. To address this challenge, future research should focus on developing and publishing tutorial articles that provide step‐by‐step guidance on their application, along with ready‐to‐use software code for platforms such as R and SAS. Such resources would facilitate a broader adoption of these methods and enhance the quality of RWD studies.

### Strengths and limitations

This review provides a comprehensive overview of existing approaches to adjust for treatment switching in RWD studies. We employed an extensive search strategy across two databases, supplemented by a snowball search, and conducted a thorough synthesis of statistical methods, detailing their assumptions, advantages, and disadvantages to support epidemiologists and clinical researchers in selecting the most appropriate approach. Nonetheless, our study has some limitations.

First, we identified 45 relevant studies, with 20 obtained through our systematic search and an additional 25 through snowballing. The relatively low number of studies retrieved through the systematic search alone likely reflects challenges in identifying relevant articles using database searches, as treatment switching methodologies are often not explicitly indexed or described in study abstracts. To address this, we employed an extensive snowballing approach, which allowed us to identify additional relevant studies missed in the initial search. This difficulty in study identification may also explain why a previous review included fewer relevant articles than our study.^69^

Second, the included studies relied on empirical and simulated data, focusing on specific settings and scenarios (e.g., varying levels of missing data and number of treatment switches). As a result, readers should be cautious when applying these methods to their data, as they may differ from the contexts and assumptions of the studies reviewed. Moreover, the inclusion of empirical data‐based studies presents a limitation in that these studies do not know the true value of the treatment effects. The lack of this information prevents these studies from assessing the relative performance of the compared methods in accurately estimating treatment effects. This prevents a direct evaluation of how well these methods perform in estimating causal relationships, as any bias or error in the estimates cannot be quantified against a “true” benchmark.

Third, our review focused on statistical approaches for treatment switching, whereas RWD with treatment switching is often also characterized by missing data. Many of the methods reviewed do not explicitly account for missing data, which can further complicate causal inference. Consequently, while the statistical methods reviewed may perform well in treatment‐switching scenarios in fully observed datasets, their performance may be compromised when applied to RWD with missing values. This underscores the need for combining robust missing data techniques, such as multiple imputation, with the methods identified to ensure reliable causal inferences when applying these methods to RWD settings, and further research into this area is warranted.

Finally, some of the included studies were empirical in nature and therefore did not assess the performance of methods in estimating treatment effects, as the true causal effect was unknown. These studies nonetheless contributed valuable information on assumptions, implementation, and contextual use. In addition, several methods described in the review (particularly traditional approaches) were not specifically designed to adjust for treatment switching but were included in the original studies as comparators. We reported these for the sake of completeness and to reflect current practices.

## CONCLUSION

The statistical methods reviewed in this study present promising strategies for addressing treatment switching in RWD studies. However, their ability to produce accurate causal estimates depends on stringent assumptions, particularly “no unmeasured confounding” and “correct model specification.” Addressing these assumptions is particularly challenging in settings with many treatment switches and high levels of missing data. To improve the accuracy of causal effect estimates in RWD studies with treatment switching, we recommend future research to explore the performance of missing data techniques in combination with the methods identified in this review. Additionally, the development and publication of tutorial articles with step‐by‐step guidance and ready‐to‐use software code would facilitate the broader adoption of these methods by research across disciplines.

## FUNDING

This work was funded by ZonMW (Grant number 10580012210010).

## CONFLICT OF INTEREST

The authors declared no competing interests for this work.

## AUTHOR CONTRIBUTIONS

R.J.C. wrote the manuscript; R.J.C., Â.J.B., M.E., J.M.D., F.L., J.B., and J.E. designed the research; R.J.C., Â.J.B., M.E., and J.M.D. performed the research; R.J.C., Â.J.B., J.M.D., J.E., and A.N.V. analyzed the data.

## Supporting information


Appendix S1.
