# Supplementary material for: Statistical Methods to Adjust for Treatment Switching in Real‐World Clinical Studies: A Scoping Review and Descriptive Comparison
Source: Clin Pharmacol Ther. 2025 Jul 23;118(5):1022–36. doi: 10.1002/cpt.70013 (PMC12598132; doi:10.1002/cpt.70013)
Supplement: Supplementary file 1 — Appendix S1. [file CPT-118-1022-s001.docx]

Table of Contents

[Appendix 1: Search strategy 2](#_Toc195017649)

[Search strings 2](#_Toc195017650)

[Excluded articles after full-text screening 4](#_Toc195017651)

[Appendix 2: Definition of treatment switching 10](#_Toc195017652)

[Appendix 3: Preferred Reporting Items for Systematic reviews and Meta-Analyses extension for Scoping Reviews (PRISMA-ScR) Checklist 11](#_Toc195017653)

# Appendix 1: Search strategy

## Search strings

#### Database(s): Ovid MEDLINE(R) ALL 1946 to December 02, 2024

| # | Searches | Results |
| --- | --- | --- |
| 1 | exp Treatment Switching/ or exp Drug Substitution/ | 4698 |
| 2 | ((treatment* or intervention* or therap* or drug* or medication* or regimen) adj3 (switch* or chang* or crossover or alter* or substitut* or replace* or conversion or adapt* or shift* or adjust*)).ti,ab,kf. | 404308 |
| 3 | (("second-line" or "second line") adj3 (treatment* or intervention* or therap* or drug* or medication* or regimen*)).ti,ab,kf. | 21999 |
| 4 | or/1-3 | 427549 |
| 5 | ((Instrumental adj2 Variables) or (Time adj1 Dependent adj2 covariates) or (marginal adj2 structural adj2 model*) or ("G-computation" or (G adj1 computation)) or (propensity score adj2 method*) or ("targeted maximum" adj2 "likelihood estimator") or (matching adj2 method*) or (mediation adj2 analys*)).ti,ab,kf. | 30848 |
| 6 | (statistic* adj3 (adjust* or correct* or "co variabl*" or covariabl* or adapt*)).ti,ab,kf. | 10975 |
| 7 | ((Inverse adj2 probability) or (censoring adj2 weight*) or (time adj2 varying adj2 covariate) or (two adj2 stage adj2 model)).ti,ab,kf. | 11627 |
| 8 | or/5-7 | 52377 |
| 9 | exp Controlled Clinical Trial/ or exp Intention to Treat Analysis/ or exp cross-over studies/ or exp double-blind method/ or exp random allocation/ or exp single-blind method/ or exp Equivalence Trial/ or exp Clinical Trial/ | 1129611 |
| 10 | (randomized or randomly or sham or placebo* or allocat* or retrospective* or prospective*).ti,ab,kf. | 3226629 |
| 11 | ((control* or multicenter* or multicentr* or crossover or "cross over" or clinical* or "intention to treat" or equivalence) adj2 (study or studies or trial*)).ti,ab,kf. | 1487258 |
| 12 | ((singl* or doubl* or tripl*) adj2 (blind* or dumm* or mask*)).ti,ab,kf. | 211631 |
| 13 | exp Cohort Studies/ or exp follow-up studies/ or exp longitudinal studies/ or exp prospective studies/ or exp retrospective studies/ or *clinical study/ | 2680077 |
| 14 | ((cohort* or longitudinal* or follow up or followup or prospective or retrospective) adj2 (stud* or design* or analy* or survey* or trial*)).ti,ab,kf. | 1505024 |
| 15 | or/9-14 | 5605826 |
| 16 | 4 and 8 and 15 | 1486 |

#### Database(s): Embase Classic+Embase 1947 to 2024 December 02

| # | Searches | Results |
| --- | --- | --- |
| 1 | exp treatment switching/ or exp drug substitution/ | 58986 |
| 2 | ((treatment* or intervention* or therap* or drug* or medication* or regimen) adj3 (switch* or chang* or crossover or alter* or substitut* or replace* or conversion or adapt* or shift* or adjust*)).ti,ab,kf. | 625423 |
| 3 | (("second-line" or "second line") adj3 (treatment* or intervention* or therap* or drug* or medication* or regimen*)).ti,ab,kf. | 40154 |
| 4 | or/1-3 | 712613 |
| 5 | ((Instrumental adj2 Variables) or (Time adj1 Dependent adj2 covariates) or (marginal adj2 structural adj2 model*) or ("G-computation" or (G adj1 computation)) or (propensity score adj2 method*) or ("targeted maximum" adj2 "likelihood estimator") or (matching adj2 method*) or (mediation adj2 analys*)).ti,ab,kf. | 36667 |
| 6 | (statistic* adj3 (adjust* or correct* or "co variabl*" or covariabl* or adapt*)).ti,ab,kf. | 16119 |
| 7 | ((Inverse adj2 probability) or (censoring adj2 weight*) or (time adj2 varying adj2 covariate) or (two adj2 stage adj2 model)).ti,ab,kf. | 16958 |
| 8 | or/5-7 | 68153 |
| 9 | exp controlled clinical trial/ or exp crossover procedure/ or exp double blind procedure/ or exp intention to treat analysis/ or exp single blind procedure/ or exp triple blind procedure/ or exp randomization/ or exp equivalence trial/ or exp clinical trial/ or exp "clinical trial (topic)"/ or exp cohort analysis/ or longitudinal study/ or prospective study/ or retrospective study/ or exp follow up/ or "major clinical study"/ | 9683684 |
| 10 | ((cohort* or longitudinal* or follow up or followup or prospective or retrospective or observation*) adj2 (stud* or design* or analy* or survey* or trial*)).ti,ab,kf. | 2482899 |
| 11 | (randomized or randomly or sham or placebo* or allocat* or retrospective* or prospective*).ti,ab,kf. | 4922654 |
| 12 | ((control* or multicenter* or multicentr* or crossover or "cross over" or clinical* or "intention to treat" or equivalence) adj2 (study or studies or trial*)).ti,ab,kf. | 2118407 |
| 13 | ((singl* or doubl* or tripl*) adj2 (blind* or dumm* or mask*)).ti,ab,kf. | 306554 |
| 14 | or/9-13 | 11548400 |
| 15 | 4 and 8 and 14 | 2937 |

## Excluded articles after full-text screening

##### Wrong design (e.g., randomized setting, applied clinical studies not comparing at least two methods, not addressing treatment switching)

1. Akushevich I, Arbeev K, Kravchenko J, Berry M. Causal Effects of Time-Dependent Treatments in Older Patients with Non-Small Cell Lung Cancer. Plos one. 2015 Apr 7;10(4):e0121406.
2. Ali AK, Hartzema AG, Winterstein AG, Segal R, Lu X, Hendeles L. Application of multicategory exposure marginal structural models to investigate the association between long-acting beta-agonists and prescribing of oral corticosteroids for asthma exacerbations in the Clinical Practice Research Datalink. Value in Health. 2015 Mar 1;18(2):260-70.
3. Alshreef A, Latimer N, Tappenden P, Dixon S. Assessing Methods for Adjusting Estimates of Treatment Effectiveness for Patient Nonadherence in the Context of Time-to-Event Outcomes and Health Technology Assessment: A Simulation Study. Medical Decision Making. 2025 Jan;45(1):60-73.
4. Artman WJ, Johnson BA, Lynch KG, McKay JR, Ertefaie A. Bayesian set of best dynamic treatment regimes: Construction and sample size calculation for SMARTs with binary outcomes. Statistics in medicine. 2022 Apr 30;41(9):1688-708.
5. Atassi N, Cudkowicz ME, Schoenfeld DA. Advanced statistical methods to study the effects of gastric tube and non-invasive ventilation on functional decline and survival in amyotrophic lateral sclerosis. Amyotrophic Lateral Sclerosis. 2011 Jul 1;12(4):272-7.
6. Bai X, Liu J, Li L, Faries D. Adaptive truncated weighting for improving marginal structural model estimation of treatment effects informally censored by subsequent therapy. Pharmaceutical Statistics. 2015 Nov;14(6):448-54.
7. Baker SG, Kramer BS, Lindeman KS. Latent class instrumental variables: a clinical and biostatistical perspective. Statistics in medicine. 2016 Jan 15;35(1):147-60.
8. Bembom O, van der Laan MJ. Analyzing sequentially randomized trials based on causal effect models for realistic individualized treatment rules. Statistics in medicine. 2008 Aug 30;27(19):3689-716.
9. Birnie K, Tomson C, Caskey FJ, Ben-Shlomo Y, Nitsch D, Casula A, Murray EJ, Sterne JA. Comparative effectiveness of dynamic treatment strategies for medication use and dosage: Emulating a target trial using observational data. Epidemiology. 2023 Nov 1;34(6):879-87.
10. Brentnall AR, Sasieni P, Cuzick J. Estimating efficacy in trials with selective crossover. Statistics in Medicine. 2017 Jul 10;36(15):2333-46.
11. Brumback BA, Hernán MA, Haneuse SJ, Robins JM. Sensitivity analyses for unmeasured confounding assuming a marginal structural model for repeated measures. Statistics in medicine. 2004 Mar 15;23(5):749-67.
12. Dodd S, Williamson P, White IR. Adjustment for treatment changes in epilepsy trials: a comparison of causal methods for time-to-event outcomes. Statistical Methods in Medical Research. 2019 Mar;28(3):717-33.
13. Evans R, Hawkins N, Dequen-O’Byrne P, McCrea C, Muston D, Gresty C, Ghate SR, Fan L, Hettle R, Abrams KR, de Bono J. Exploring the impact of treatment switching on overall survival from the PROfound study in homologous recombination repair (HRR)-mutated metastatic castration-resistant prostate cancer (mCRPC). Targeted Oncology. 2021 Sep;16:613-23.
14. Feliciano JL, McLoone D, Xu Y, Quek RG, Kuznik A, Pouliot JF, Gullo G, Rietschel P, Guyot P, Konidaris G, Chan K. Impact of the treatment crossover design on comparative efficacy in EMPOWER-Lung 1: Cemiplimab monotherapy as first-line treatment of advanced non-small cell lung cancer. Frontiers in Oncology. 2023 Apr 4;12:1081729.
15. Fisher VA, Comment LA. Multi-state survival models with treatment effects and biomarkers: Simulations for study design assessment. Cancer Epidemiology. 2022 Dec 1;81:102272.
16. Ford D, Robins JM, Petersen ML, Gibb DM, Gilks CF, Mugyenyi P, Grosskurth H, Hakim J, Katabira E, Babiker AG, Walker AS. The impact of different CD4 cell-count monitoring and switching strategies on mortality in HIV-infected African adults on antiretroviral therapy: an application of dynamic marginal structural models. American journal of epidemiology. 2015 Oct 1;182(7):633-43.
17. Fukuda M, Sakamaki K, Oba K. The net benefit for time-to-event outcome in oncology clinical trials with treatment switching. Clinical Trials. 2023 Dec;20(6):670-80.
18. Ionescu‐Ittu R, Delaney JA, Abrahamowicz M. Bias–variance trade‐off in pharmacoepidemiological studies using physician‐preference‐based instrumental variables: a simulation study. Pharmacoepidemiology and drug safety. 2009 Jul;18(7):562-71.
19. Godin O, Elbejjani M, Kaufman JS. Body mass index, blood pressure, and risk of depression in the elderly: a marginal structural model. American journal of epidemiology. 2012 Aug 1;176(3):204-13.
20. Gorrod HB, Latimer NR, Damian D, Hettle R, Harty GT, Wong SL. Impact of nonrandomized dropout on treatment switching adjustment in the relapsing–remitting multiple sclerosis CLARITY trial and the CLARITY extension study. Value in Health. 2019 Jul 1;22(7):772-6.
21. Graffeo N, Latouche A, Le Tourneau C, Chevret S. ipcwswitch: an R package for inverse probability of censoring weighting with an application to switches in clinical trials. Computers in biology and medicine. 2019 Aug 1;111:103339.
22. Hernán MA, Hernández-Díaz S. Beyond the intention-to-treat in comparative effectiveness research. Clinical trials. 2012 Feb;9(1):48-55.
23. Ishak KJ, Proskorovsky I, Korytowsky B, Sandin R, Faivre S, Valle J. Methods for adjusting for bias due to crossover in oncology trials. Pharmacoeconomics. 2014 Jun;32:533-46.
24. Karim ME, Gustafson P, Petkau J, Zhao Y, Shirani A, Kingwell E, Evans C, Van Der Kop M, Oger J, Tremlett H. Marginal structural Cox models for estimating the association between β-interferon exposure and disease progression in a multiple sclerosis cohort. American journal of epidemiology. 2014 Jul 15;180(2):160-71.
25. Kasza J, Polkinghorne KR, Marshall MR, McDonald SP, Wolfe R. Clustering and residual confounding in the application of marginal structural models: dialysis modality, vascular access, and mortality. American journal of epidemiology. 2015 Sep 15;182(6):535-43.
26. Khanal N, Marshall MR, Ma TM, Pridmore PJ, Williams AB, Rankin AP. Comparison of outcomes by modality for critically ill patients requiring renal replacement therapy: a single-centre cohort study adjusting for time-varying illness severity and modality exposure. Anaesthesia and intensive care. 2012 Mar;40(2):260-8.
27. Kidwell KM, Ko JH, Wahed AS. Inference for the median residual life function in sequential multiple assignment randomized trials. Statistics in medicine. 2014 Apr 30;33(9):1503-13.
28. Ko H, Hogan JW, Mayer KH. Estimating causal treatment effects from longitudinal HIV natural history studies using marginal structural models. Biometrics. 2003 Mar;59(1):152-62.
29. Latimer NR, Abrams KR, Amonkar MM, Stapelkamp C, Swann RS. Adjusting for the confounding effects of treatment switching—the BREAK-3 trial: dabrafenib versus dacarbazine. The oncologist. 2015 Jul 1;20(7):798-805.
30. Latimer NR, Abrams KR, Lambert PC, Crowther MJ, Wailoo AJ, Morden JP, Akehurst RL, Campbell MJ. Adjusting survival time estimates to account for treatment switching in randomized controlled trials—an economic evaluation context: methods, limitations, and recommendations. Medical Decision Making. 2014 Apr;34(3):387-402.
31. Latimer NR, Abrams KR, Lambert PC, Crowther MJ, Wailoo AJ, Morden JP, Akehurst RL, Campbell MJ. Adjusting for treatment switching in randomised controlled trials–a simulation study and a simplified two-stage method. Statistical methods in medical research. 2017 Apr;26(2):724-51.
32. Latimer NR, Abrams KR, Lambert PC, Morden JP, Crowther MJ. Assessing methods for dealing with treatment switching in clinical trials: a follow-up simulation study. Statistical methods in medical research. 2018 Mar;27(3):765-84.
33. Latimer NR, Abrams KR, Siebert U. Two-stage estimation to adjust for treatment switching in randomised trials: a simulation study investigating the use of inverse probability weighting instead of re-censoring. BMC medical research methodology. 2019 Dec;19:1-9.
34. Latimer NR, Bell H, Abrams KR, Amonkar MM, Casey M. Adjusting for treatment switching in the METRIC study shows further improved overall survival with trametinib compared with chemotherapy. Cancer Medicine. 2016 May;5(5):806-15.
35. Latimer NR, Dewdney A, Campioni M. A cautionary tale: an evaluation of the performance of treatment switching adjustment methods in a real world case study. BMC Medical Research Methodology. 2024 Jan 22;24(1):17.
36. Latimer NR, Henshall C, Siebert U, Bell H. Treatment switching: statistical and decision-making challenges and approaches. International journal of technology assessment in health care. 2016 Jan;32(3):160-6.
37. Latimer NR, White IR, Tilling K, Siebert U. Improved two-stage estimation to adjust for treatment switching in randomised trials: g-estimation to address time-dependent confounding. Statistical methods in medical research. 2020 Oct;29(10):2900-18.
38. Lavori PW, Dawson R. Improving the efficiency of estimation in randomized trials of adaptive treatment strategies. Clinical Trials. 2007 Aug;4(4):297-308.
39. Li L, Tang S, Jiang L. On an enhanced rank‐preserving structural failure time model to handle treatment switch, crossover, and dropout. Statistics in Medicine. 2017 May 10;36(10):1532-47.
40. Li Z, Valenstein M, Pfeiffer P, Ganoczy D. A global logrank test for adaptive treatment strategies based on observational studies. Statistics in medicine. 2014 Feb 28;33(5):760-71.
41. Madsen JE, Scheike T, Pipper C. Unbiased and efficient estimation of causal treatment effects in crossover trials. Biometrical Journal. 2023 Dec;65(8):2200170.
42. Mahaffey KW, Pieper KS, Lokhnygina Y, Califf RM, Antman EM, Kleiman NS, Goodman SG, White HD, Rao SV, Hochman JS, Cohen M. The impact of postrandomization crossover of therapy in acute coronary syndromes care. Circulation: Cardiovascular Quality and Outcomes. 2011 Mar;4(2):211-9.
43. Marcus SM, Gibbons RD. Estimating the efficacy of receiving treatment in randomized clinical trials with noncompliance. Health Services and Outcomes Research Methodology. 2001 Dec;2:247-58.
44. Michiels H, Sotto C, Vandebosch A, Vansteelandt S. A novel estimand to adjust for rescue treatment in randomized clinical trials. Statistics in Medicine. 2021 Apr 30;40(9):2257-71.
45. Miyahara S, Wahed AS. Weighted Kaplan–Meier estimators for two‐stage treatment regimes. Statistics in medicine. 2010 Nov 10;29(25):2581-91.
46. Petersen ML, Deeks SG, Martin JN, Van Der Laan MJ. History-adjusted marginal structural models for estimating time-varying effect modification. American Journal of Epidemiology. 2007 Nov 1;166(9):985-93.
47. Penning de Vries BB, Groenwold RH. Bias of time‐varying exposure effects due to time‐varying covariate measurement strategies. Pharmacoepidemiology and Drug Safety. 2022 Jan;31(1):22-7.
48. Pullenayegum EM, Lam C, Manlhiot C, Feldman BM. Fitting marginal structural models: estimating covariate-treatment associations in the reweighted data set can guide model fitting. Journal of clinical epidemiology. 2008 Sep 1;61(9):875-81.
49. Schaubel DE, Wolfe RA, Port FK. A sequential stratification method for estimating the effect of a time-dependent experimental treatment in observational studies. Biometrics. 2006 Sep;62(3):910-7.
50. Seaman SR, Keogh RH. Simulating data from marginal structural models for a survival time outcome. Biometrical Journal. 2024 Dec;66(8):e70010.
51. Skaltsa K, Ivanescu C, Naidoo S, Phung D, Holmstrom S, Latimer NR. Adjusting overall survival estimates after treatment switching: a case study in metastatic castration-resistant prostate cancer. Targeted Oncology. 2017 Feb;12:111-21.
52. Smith AR, Zhu D, Goodrich NP, Merion RM, Schaubel DE. Estimating the effect of a rare time‐dependent treatment on the recurrent event rate. Statistics in medicine. 2018 May 30;37(12):1986-96.
53. Soltanian AR, Faghihzadeh S, Gerami A, Mehdibarzi D, Jing Cheng J. Estimation of Treatment Effects in Crossover Clinical Trials with Noncompliance. Iranian Journal of Epidemiology. 2010 Jun 10;6(1):18-25.
54. Sussman JB, Hayward RA. An IV for the RCT: using instrumental variables to adjust for treatment contamination in randomised controlled trials. Bmj. 2010 May 4;340.
55. Vansteelandt S, Sjolander A. Revisiting g-estimation of the effect of a time-varying exposure subject to time-varying confounding. Epidemiologic Methods. 2016 Dec 1;5(1):37-56.
56. Watkins C, Huang X, Latimer N, Tang Y, Wright EJ. Adjusting overall survival for treatment switches: commonly used methods and practical application. Pharmaceutical statistics. 2013 Nov;12(6):348-57.
57. Watson JA, Holmes CC. Machine learning analysis plans for randomised controlled trials: detecting treatment effect heterogeneity with strict control of type I error. Trials. 2020 Dec;21:1-0.
58. Xu Q, Przepiorka D. Using marginal structural models to analyze the impact of subsequent therapy on the treatment effect in survival data: Simulations and clinical trial examples. Pharmaceutical Statistics. 2021 Nov;20(6):1088-101.
59. Xu J, Liu G, Wang B. Bias and Type I error Control in Correcting Treatment Effect for Treatment Switching Using Marginal Structural Models in Phase III Oncology Trials. Journal of Biopharmaceutical Statistics. 2022 Nov 2;32(6):897-914.
60. Xu Y, Müller P, Wahed AS, Thall PF. Bayesian nonparametric estimation for dynamic treatment regimes with sequential transition times. Journal of the American Statistical Association. 2016 Jul 2;111(515):921-50.
61. Xu Y, Wu M, He W, Liao Q, Mai Y. Teasing out the overall survival benefit with adjustment for treatment switching to multiple treatments. Statistics in Biopharmaceutical Research. 2022 Oct 2;14(4):592-601.
62. Yamaguchi T, Ohashi Y. Adjusting for differential proportions of second‐line treatment in cancer clinical trials. Part I: structural nested models and marginal structural models to test and estimate treatment arm effects. Statistics in Medicine. 2004 Jul 15;23(13):1991-2003.
63. Yu Y, Zhang M, Mukherjee B. An inverse probability weighted regression method that accounts for right‐censoring for causal inference with multiple treatments and a binary outcome. Statistics in Medicine. 2023 Sep 10;42(20):3699-715.
64. Zhang M, Wang Y. Adjusting for observational secondary treatments in estimating the effects of randomized treatments. Biostatistics. 2013 Jul 1;14(3):491-501.
65. Zheng W, Luo Z, Van Der Laan MJ. Marginal structural models with counterfactual effect modifiers. The international journal of biostatistics. 2018 Jun 26;14(1):20180039.

##### Wrong publication type (e.g., guideline recommendations, poster abstract)

1. Desai RJ, Kim SC, Curtis JR, Bosco JL, Eichelberger B, Barr CE, Lockhart CM, Bradbury BD, Clewell J, Cohen HP, Gagne JJ. Methodologic considerations for noninterventional studies of switching from reference biologic to biosimilars. Pharmacoepidemiology and Drug Safety. 2020 Jul;29(7):757-69.
2. Barbulescu A, Sjölander A, Delcoigne B, Askling J, Frisell T. POS0379 WHEN SHOULD WE USE MARGINAL STRUCTURAL MODELS FOR DRUG STUDIES IN RHEUMATOLOGY? THE EXAMPLE OF CORTICOSTEROIDS AND INFECTIONS IN RHEUMATOID ARTHRITIS. Annals of the Rheumatic Diseases. 2023 Jun 1;82:443.

# Appendix 2: Definition of treatment switching

*Treatment switching* refers to deviations from an initially assigned treatment strategy during the follow-up period of a clinical study. In real-world observational settings, patients may discontinue or change treatments over time, often in response to evolving health status, treatment response, side effects, and/or physician judgment.

These factors can act as *time-varying confounders*: variables that (1) change over time, (2) influence both future treatment decisions and the outcome, and, in the context of treatment switching, (3) may themselves be affected by earlier treatment. When this occurs, conventional statistical methods (e.g., standard regression or baseline adjustment) may yield biased estimates, because they do not appropriately handle situations where earlier treatment influences confounders that, in turn, affect both subsequent treatment and the outcome.

To illustrate, consider a simplified treatment trajectory over two time points:

- L_0_: a baseline confounder measured at baseline (time t₀)
- A₀: treatment assigned or initiated at baseline (t₀)
- L₁: a time-varying confounder measured at the next time point (t₁)
- A₁: treatment at t₁, possibly reflecting a switch or continuation
- Y: the outcome measured at the end of follow-up

In this structure, A₀ may influence L₁, which in turn affects both A₁ and Y. Because L₁ is influenced by prior treatment (A₀) and also affects future treatment (A₁) and the outcome (Y), it acts as a time-varying confounder affected by prior treatment.

Adjusting for L₁ using standard regression or baseline-adjusted methods may lead to biased estimates. This is because L₁ lies on a causal pathway from A₀ to Y, and conditioning on it blocks part of that pathway, distorting the estimate of the treatment effect. This structure is represented in the following figure:


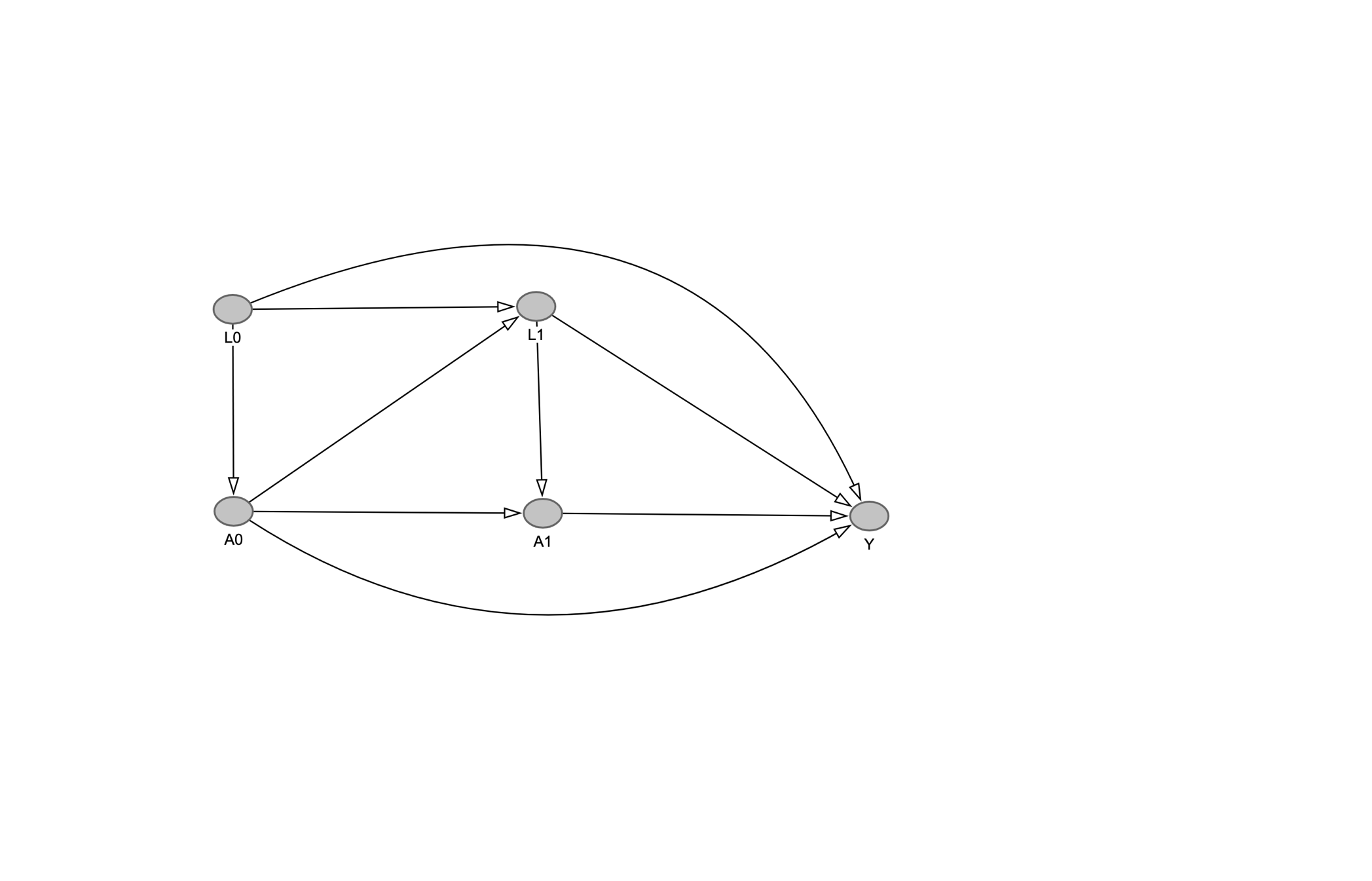


Figure: Graphical representation of a simplified example of treatment switching

# Appendix 3: Preferred Reporting Items for Systematic reviews and Meta-Analyses extension for Scoping Reviews (PRISMA-ScR) Checklist

| **SECTION** | | **ITEM** | | **PRISMA-ScR CHECKLIST ITEM** | | **REPORTED**  **ON PAGE #** | |
| --- | --- | --- | --- | --- | --- | --- | --- |
| **TITLE** | | | | | | | |
| Title | | 1 | | Identify the report as a scoping review. | | 1 | |
| **ABSTRACT** | | | | | | | |
| Structured summary | | 2 | | Provide a structured summary that includes (as applicable): background, objectives, eligibility criteria, sources of evidence, charting methods, results, and  conclusions that relate to the review questions and objectives. | | 2 | |
| **INTRODUCTION** | | | | | | | |
| Rationale | | 3 | | Describe the rationale for the review in the context of what is already known. Explain why the review questions/objectives lend themselves to a scoping review approach. | | 3-4 | |
| Objectives | | 4 | | Provide an explicit statement of the questions and objectives being addressed with reference to their key elements (e.g., population or participants, concepts, and  context) or other relevant key elements used to conceptualize the review questions and/or objectives. | | 3-4 | |
| **METHODS** | | | | | | | |
| Protocol and registration | | 5 | | Indicate whether a review protocol exists; state if and where it can be accessed (e.g., a Web address); and if available, provide registration information, including the registration number. | | 4 | |
| Eligibility criteria | | 6 | | Specify characteristics of the sources of evidence used as eligibility criteria (e.g., years considered, language,  and publication status), and provide a rationale. | | 5 | |
| Information sources* | | 7 | | Describe all information sources in the search (e.g., databases with dates of coverage and contact with authors to identify additional sources), as well as the date the most recent search was executed. | | 4-5 | |
| Search | | 8 | | Present the full electronic search strategy for at least 1  database, including any limits used, such that it could be repeated. | | Appendix 1 | |
| Selection of sources of evidence† | | 9 | | State the process for selecting sources of evidence (i.e., screening and eligibility) included in the scoping review. | | 5 | |
| Data charting process‡ | | 10 | | Describe the methods of charting data from the included sources of evidence (e.g., calibrated forms or forms that have been tested by the team before their use, and whether data charting was done independently or in  duplicate) and any processes for obtaining and confirming data from investigators. | | 5-6 | |
| Data items | | 11 | | List and define all variables for which data were sought and any assumptions and simplifications made. | | 5-6 | |
| Critical appraisal of individual sources of evidence§ | | 12 | | If done, provide a rationale for conducting a critical appraisal of included sources of evidence; describe the  methods used and how this information was used in any data synthesis (if appropriate). | | NA | |
| Synthesis of results | | 13 | | Describe the methods of handling and summarizing the data that were charted. | | 6 | |
| **SECTION** | | **ITEM** | | **PRISMA-ScR CHECKLIST ITEM** | | **REPORTED**  **ON PAGE #** | |
| **RESULTS** | | | | | | | |
| Selection of sources of evidence | | 14 | | Give numbers of sources of evidence screened, assessed for eligibility, and included in the review, with  reasons for exclusions at each stage, ideally using a flow diagram. | | 7, figure 1 | |
| Characteristics of sources of evidence | | 15 | | For each source of evidence, present characteristics for which data were charted and provide the citations. | | 7, Table 1 | |
| Critical appraisal within sources of evidence | | 16 | | If done, present data on critical appraisal of included sources of evidence (see item 12). | | NA | |
| Results of  individual sources of evidence | | 17 | | For each included source of evidence, present the  relevant data that were charted that relate to the review questions and objectives. | | 7-20, Table 1 | |
| Synthesis of results | | 18 | | Summarize and/or present the charting results as they relate to the review questions and objectives. | | 7-20 | |
| **DISCUSSION** | | | | | | | |
| Summary of evidence | | 19 | | Summarize the main results (including an overview of concepts, themes, and types of evidence available), link to the review questions and objectives, and consider the relevance to key groups. | | 21 | |
| Limitations | | 20 | | Discuss the limitations of the scoping review process. | | 24-25 | |
| Conclusions | | 21 | | Provide a general interpretation of the results with respect to the review questions and objectives, as well  as potential implications and/or next steps. | | 25 | |
| **FUNDING** | | | | | | | |
| Funding | | 22 | | Describe sources of funding for the included sources of evidence, as well as sources of funding for the scoping review. Describe the role of the funders of the scoping review. | | 1 | |

JBI = Joanna Briggs Institute; PRISMA-ScR = Preferred Reporting Items for Systematic reviews and Meta-Analyses extension for Scoping Reviews.

* Where *sources of evidence* (see second footnote) are compiled from, such as bibliographic databases, social media platforms, and Web sites.

† A more inclusive/heterogeneous term used to account for the different types of evidence or data sources (e.g., quantitative and/or qualitative research, expert opinion, and policy documents) that may be eligible in a scoping review as opposed to only studies. This is not to be confused with *information sources* (see first footnote).

‡ The frameworks by Arksey and O’Malley (6) and Levac and colleagues (7) and the JBI guidance (4, 5) refer to the process of data extraction in a scoping review as data charting*.*

§ The process of systematically examining research evidence to assess its validity, results, and relevance before using it to inform a decision. This term is used for items 12 and 19 instead of "risk of bias" (which is more applicable to systematic reviews of interventions) to include and acknowledge the various sources of evidence that may be used in a scoping review (e.g., quantitative and/or qualitative research, expert opinion, and policy document).

*From:* Tricco AC, Lillie E, Zarin W, O'Brien KK, Colquhoun H, Levac D, et al. PRISMA Extension for Scoping Reviews (PRISMA- ScR): Checklist and Explanation. Ann Intern Med. ;169:467–473. doi: 10.7326/M18-0850
